# *PRNP* Polymorphisms in Eight Local Goat Populations/Breeds from Central and Southern Italy

**DOI:** 10.3390/ani11020333

**Published:** 2021-01-28

**Authors:** Martina Torricelli, Carla Sebastiani, Marcella Ciullo, Simone Ceccobelli, Barbara Chiappini, Gabriele Vaccari, Antonio Capocefalo, Michela Conte, Samira Giovannini, Emiliano Lasagna, Francesca Maria Sarti, Massimo Biagetti

**Affiliations:** 1 Istituto Zooprofilattico Sperimentale dell’Umbria e delle Marche-Togo Rosati (IZSUM), Via Salvemini 1, 06126 Perugia, Italy; m.torricelli@izsum.it (M.T.); c.sebastiani@izsum.it (C.S.); m.ciullo@izsum.it (M.C.); 2 Dipartimento di Scienze Agrarie, Alimentari e Ambientali, Università Politecnica delle Marche, 60131 Ancona, Italy; s.ceccobelli@staff.univpm.it; 3 Dipartimento di Sicurezza Alimentare, Nutrizione e Sanità Pubblica Veterinaria, Istituto Superiore di Sanità, 00161 Rome, Italy; barbara.chiappini@iss.it (B.C.); gabriele.vaccari@iss.it (G.V.); antonio.capocefalo@iss.it (A.C.); michela.conte@iss.it (M.C.); 4 Dipartimento di Scienze Agrarie, Alimentari e Ambientali, University of Perugia, 06121 Perugia, Italy; samira.giovannini@gmail.com (S.G.); emiliano.lasagna@unipg.it (E.L.); francesca.sarti@unipg.it (F.M.S.)

**Keywords:** goats, SNPs, *PRNP*, scrapie, genetic selection, local populations/breeds

## Abstract

**Simple Summary:**

Scrapie is a naturally occurring transmissible spongiform encephalopathy of sheep and goats. Polymorphisms in single nucleotides in the coding sequence of the prion protein gene play a major role in relative susceptibility or resistance to classical scrapie. The most recent modification of European Union Regulation 999/2001 (Regulation EU 772/2020) deals with the possible use of genetics in goats for scrapie outbreak management. In this work, sequence analysis of the prion protein gene was performed, with the main aim to obtain information about the genetic variability of eight Italian local populations/breeds and establishing phylogenetic relationships between the breeds/populations. This research could help to promote the adoption of selective breeding programs as a possible future strategy in scrapie control and outbreak prevention. In particular, genomic DNA was extracted from 219 goat whole blood samples and 13 polymorphic sites were observed, giving 24 different alleles. We observed the K222 allele, associated to scrapie resistance, with a mean frequency of 10%. Interestingly, we also described in Italy the circulation of the S146 allele: In particular, S146 only segregated in the Alpine breed at a frequency of 3.7%. This study adds information on genetic variability of the *PRNP* locus in goat populations/breeds, so contributing to the design of genetic control measure both in scrapie outbreaks and in disease prevention.

**Abstract:**

In goats, as in sheep, genotypes of the prion protein gene (*PRNP*) can influence animals’ susceptibility to scrapie. Since the polymorphic codons in sheep are well known, a genetic selection plan has been implemented in Europe, in order to reduce the prevalence of susceptible genotypes to scrapie. In Italy, no breeding plan for scrapie resistance in goats has been adopted, yet. Likewise, according to the most recent modification of Regulation EU 999/2001 (Regulation EU 772/2020) of the European Commission (EU), based on all the available experimental and in field data, K222, D146 and S146 polymorphisms could be used as scrapie resistance alleles in genetic management both in scrapie outbreaks and in disease prevention. In order to collect data on the variability of *PRNP*, the present study aimed to analyze the sequence of the *PRNP* gene in eight Italian local goat populations/breeds reared in central and southern Italy (Bianca Monticellana, Capestrina, Facciuta della Valnerina, Fulva del Lazio, Garganica, Grigia Ciociara, Grigia Molisana, and Teramana), some of which were investigated for the first time; moreover, two cosmopolitan breeds (Alpine and Saanen) were included. Blood samples were collected from 219 goats. Genomic DNA was extracted from whole blood. DNA was used as template in PCR amplification of the entire *PRNP* open reading frame (ORF). Purified amplicons have been sequenced and aligned to *Capra hircus PRNP.* Particularly, the alleles carrying the resistance-related 222 K polymorphism occurred in all populations with a frequency between 2.5% and 12.5%. An additional resistance allele carrying the S146 variant was observed with a frequency of 3.7% only in the Alpine breed. For three of the estimated alleles, we could not establish if the found double polymorphisms in heterozygosis were in phase, due to technical limitations. In this context, in addition to selective culling in scrapie outbreaks according to the European regulation in force, in the future, selection plans could be adopted to deal with scrapie and to control its diffusion, meanwhile paying attention to preserve a high variability of *PRNP*.

## 1. Introduction

Scrapie in sheep and goats belongs to a group of diseases known as transmissible spongiform encephalopathies or prion diseases. They represent a family of transmissible slowly progressive and invariably fatal neurodegenerative diseases affecting humans and animals [1]. To date, three different scrapie forms were found: classical scrapie, Nor98/atypical scrapie, and one case of CH1641 scrapie. Furthermore, classical scrapie was found in two variants defined CS-1 and CS-2 (mainly in Italy), which differed in proteolytic resistance of the PrP [2,3,4]. Classical scrapie, which affects all the small ruminant breeds, is spread all over the world with the exception of Australia and New Zealand, where the disease has been eradicated [5]. Susceptibility or resistance to scrapie in sheep and goats depends on the PrP genotype of the host and on the infectious strain [6,7,8]. Although the zoonotic potential of some scrapie strains has been suggested in various studies using humanized transgenic mice and cynomolgus macaques as recipients [9,10], nowadays, there is no evidence of risk for the human population as reported by European Food Safety Authority (EFSA) [11].

In sheep, three polymorphic codons of the *PRNP* (codons 136, 154, and 171) give rise to five main different alleles, namely VRQ, ARQ, AHQ, ARH, and ARR (defined with the IUPAC amino acid codes). The VRQ/VRQ, VRQ/ARQ, and ARQ/ARQ genotypes are associated with high susceptibility to classical scrapie [12], while the ARR-containing genotypes, with the exception of VRQ/ARR, have been associated with various levels of resistance to disease [13]. On this basis, according to European Union Decision 2003/100/EC (EU 2003) [14], a selection breeding program to increase the frequency of the resistance-associated ARR allele in sheep populations has been adopted by each member state in the European Community.

The breeding plans for sheep led to a frequency increase of the resistance allele. In particular, among the 13 countries characterized by a consistent number of cases, the trend analysis shows a statistically significant decrease of classical scrapie even if with certainty only for six of them [15].

As already done in sheep, single nucleotide polymorphisms (SNPs) of *PRNP* in goats could represent an opportunity to classify caprine genotypes in those susceptible or resistant to scrapie [6,16]. Furthermore, even if, to date, in accordance with the Regulation (EU) 772/2020 in force [17], genotyping in goats can be carried out by each single member State, in order to conduct selective culling of classical scrapie-susceptible animals, the possible development and adoption of genetic selection plans also for goats could play a key role in feasible and successful preventive measures for scrapie control. Many European studies showed that allele variation and polymorphisms of the *PRNP* gene can determine the susceptibility or resistance to classical scrapie in goats, but only a few of them have been proven to be associated with resistance [18]. In particular, at least 50 *PRNP* polymorphisms have been described in goat breeds [19,20], but their involvement in the modulation of the incubation times and in susceptibility to classical scrapie is less defined than in sheep [13,21]. Furthermore, another *PRNP* variant that contains three repeats of an octapeptide instead of the usual five repeats was found by Goldman [22]. Amino acid substitutions that may affect susceptibility to classical scrapie in goats include G127S (rs268292978) [23], I142M (rs268292980) [24,25], H143R (rs667226700) [26], N146S/D [27,28], R154H (rs268292981) [26,27,29,30,31], Q211R (rs268292982) [30,32], Q222K (rs268292983) [29,30,33], and S240P (rs268292984) [24,26,29,33]. In particular, the S127 and M142 polymorphisms seem to prolong the incubation period in experimental studies [23,24,25] but not to confer resistance to classical scrapie [34]. On the other hand, the R143, H154, and Q211 polymorphisms seem to be associated with resistance to classical scrapie [26,29,30,32,35]. In contrast, the H154 allele was clearly identified as being a risk factor for atypical scrapie (Nor98) [36], as it is for sheep [37].

On the other hand, the study on the genetic susceptibility to scrapie in goats conducted until now showed that the variants N146S, N146D, and Q222K are the major candidates for genetic selection, since they definitely characterize animals as being resistant to classical scrapie [38]. The results obtained for S146 and D146 highlighted a strong resistance to classical scrapie (at the same level as ARR in sheep) but not as strong as that of allele K222 [39]. Even if these results have been mainly produced in Cyprus and no data from field studies regarding resistance of alleles S146 and D146 in many European countries are available (due to absence or their low frequency), up to now, EFSA considered these alleles as resistant [39]. In addition, it has been shown that the K222 allele is associated with resistance to classical scrapie and bovine and caprine BSE [3,25,29,33,40,41].

Different and, for some aspects, contradictory results about the polymorphism at codon 240 were obtained, in particular a positive association with scrapie infection of P240 [33], or a positive association with clinical disease of S240 [34], or no association [24,26,29] or a protective effect of P240 in association with M142 in heterozygotes [30] were found. Even if the specific polymorphism at codon 240 seems not to influence scrapie susceptibility in general, it could exert an effect maybe in relation to the involved scrapie strain [40].

Since an association of the *PRNP* polymorphisms with classical or atypical scrapie susceptibility and with disease progression in ovine and caprine was established, worldwide, the study of the allele frequencies of the major variants became the main objective to be evaluated [42,43].To date, in the Italian goat populations, SNPs at codons G37V, P42P, T110P, G127S, L133Q, M137I, S138S, I142M, I42T, H143R, R151H, P168Q, T194P, T202T, R211Q, T219T, Q222K, G232G, and S240P were found, but only some of them are associated with known clinical effects [20].

On this basis, the aim of our research was to examine the *PRNP* polymorphisms for classical scrapie in eight different local goat populations/breeds reared in central and southern Italy: Bianca Monticellana (BM), Capestrina (CP), Facciuta della Valnerina (FV), Fulva del Lazio (FL), Garganica (GA), Grigia Ciociara (GC), Grigia Molisana (GM), and Teramana (TE). Some of them (GA, GC, GM, and TE) are listed in the FAO global data bank for farm animal genetic resources and are classified as endangered [44,45] (Supplementary File S1). So far, no data are available on the susceptibility of these goat populations to classical scrapie. Moreover, also two cosmopolitan breeds, that is Alpine (AL) and Saanen (SA), were included in the samples. Referring to available experimental and in-field data, K222, D146, and S146 represent the alleles that seem to confer genetic resistance to classical scrapie strains, the presence of which was registered in goat populations in the EU. Furthermore, this work goes well with the current Regulation EU 772/2020 [17] that, referring to EFSA opinion, allows European member states to adopt selective culling based on *PRNP* genotyping as an alternative to stamping out and as a valid approach to control scrapie disease in the presence of outbreaks. Indeed, in Italy, as in the other European countries, no selection plans for scrapie resistance in goats have been developed until now and the disease is still dealt with by the culling of animals in the herd affected by the outbreak. Therefore, data about the distribution of the allele variants of *PRNP* in goats obtained from this investigation could contribute to direct breeding programs to reduce the possible risk of scrapie.

## 2. Materials and Methods

### 2.1. Samples Collection

Blood samples were collected from 219 goats of different populations/breeds, belonging to different herds of central Italy, in particular the following, were sampled: 24 BM, 20 CP, 18 FV, 21 FL, 24 GA, 24 GC, 18 GM, 24 TE, 27 AL, and 19 SA. The animals were randomly sampled in the flock because genealogical information was not available. The general criterion followed in the study was based on a sample stratification considering the size of the farm and trying to take, proportionally, the same percentage of animals in all the farms. The aim of this type of sampling was to reach a representativeness of the studied population. In some case, a single sample was taken from those herds with few animals. The populations/breeds object of this study include few goats in the Italian territory and therefore the collected samples can allow a suitable representativeness to be obtained (Supplementary Table S2). Samples were collected in tubes with EDTA as anticoagulant and were stored at −20 °C until DNA extraction. No ethical approval was required, in compliance with the European Directive 2010/63/UE and the Italian Regulation D. Lgs n. 26/2014: indeed, for the purpose of the study, aliquots of blood samples, taken during obligatory routine animal sanitary controls by an authorized veterinarian, were used.

### 2.2. PRNP Gene Sequencing

Genomic DNA was extracted from whole blood using the High Pure PCR Template Preparation Kit (Roche Life Science, Mannheim, Germany) following the manufacturer’s instructions.

In total, 100 ng of extracted DNA were used as a template in PCR amplification of the entire open reading frame of the *PRNP* gene (771 bp). Amplification was performed in a 50-µL reaction volume containing 1.5 mM MgCl_2_, 200 µM dNTPs (GE Healthcare, Buckinghamshire, England), 0.8 µM of forward and reverse primers p8 (5′-CAGGTTAACGATGGTGAAAAGCCACATAGG-3′) and p9 (5′-GGAATTCTATCCTACTATGAGAAAAATGAGG-3′) (Thermo Fisher Scientific, Waltham, MA) [46], and 1 U of AmpliTaq Gold™ (Thermo Fisher Scientific). The samples were amplified with the following thermal profile: 95 °C × 15 min followed by 40 cycles at 95 °C × 1 min, 64 °C × 1 min, 72 °C × 1 min, and a final extension at 72 °C × 10 min using a Mastercycler Ep Gradient (Eppendorf AG, Hamburg, Germany). PCR products were analyzed by 2% agarose gel electrophoresis and purified with a QIAquick^®^ PCR Purification Kit (Qiagen^®^, Hilden, Germany), according to the manufacturer’s instructions.

The quantity and quality of the PCR products were determined photometrically using a Biophotometer (Eppendorf).

Purified amplicons were sequenced in both directions using about 80 ng of DNA. Sequencing reactions were performed with 3.2 pmol of primer p8 and p9 using a Big Dye Terminator Cycle Sequencing Kit v3.1 (Thermo Fisher Scientific) and detected with an ABI PRISM^®^ 3500 Genetic Analyzer (Thermo Fisher Scientific). Sequences were aligned to the *Capra hircus* gene for *PRNP* (Accession number NM_001314247.1) with ClustalW tool of BioEdit 7.0.9 software [47]. In addition, electropherograms were checked at each investigated mutation point to identify heterozygous peaks.

### 2.3. Statistical Analysis

Genotypic and allelic frequencies regarding codons 37, 110, 127, 137, 142, 146, 154, 168, 194, 211, 215, 222, and 240 were calculated within and across breed, based on counting the respective genotypes of individual animals. The Hardy–Weinberg equilibrium state was examined at each codon and breed using a chi-square test (*p* ≤ 0.05):

(1) $$X^{2}=\sum \frac{{\left(O-E\right)}^{2}}{E}$$

where O is the observed number of each genotype; E is the expected number of each genotype assuming Hardy–Weinberg equilibrium; and Σ is the summation over all possible genotypes [48,49] (R software v4.0.3). This type of statistical analysis is very important in selection studies in order to evaluate the possible inbreeding, the possible transmission of unfavorable linked traits, and in order to assess the genetic variability conservation.

The most probable *PRNP* alleles were computed on the allele frequencies observed in the populations/breeds object of this study, through PHASE v2.1 software, based on statistical inference so on probabilistic events [50] and cited in the output as “best reconstruction”.

Moreover, the genetic distances among the studied breeds were estimated on the basis of *PRNP* allele frequencies using the fixation index F_ST_ and Arlequin v3.5 software (AMOVA or Analysis of MOlecular VAriance) [51]; the pairwise F_ST_ distance matrix between populations graphic was visualized by SplitsTree4 software v4.16.2 [52]. Significant deviations from the null hypothesis were tested with 1000 permutations. The level of statistical significance was set at *p* = 0.001.

## 3. Results

Genetic variation that generates amino acid substitutions was observed in 13 codons (Table 1); the amino acids are cited as one conventional letter code (e.g., glycine: G) according to IUPAC nomenclature [53] and the variants were reported in the gray background.

The substitutions in the total sample (TOT) ranged from 0.01 to 1.00, but at the codon P240, a frequency of 0.51 for the alternative allele was estimated. At the single populations/breeds level, the highest frequencies were registered for the non-reference alleles; in particular for P194 and R215, a frequency of 0.21 in GC, and for P240, a frequency of 0.50 in BM, FL, and GC; 0.61 in FV; and 0.75 in TE. The frequency of T142 was 0.10 in the Alpine breed.

All polymorphic sites were in HW equilibrium except for codons 142 and 240, in particular the codon 142 in AL, the codon 240 in a cosmopolitan breed (SA) and in five local populations/breeds (BM, CP, FV, FL, GA), the codons 110 in CP and 222 in FV. The *PRNP* analysis in the eight populations/breeds showed high polymorphic variation; among the analyzed SNPs, 13 polymorphic sites were identified giving rise to 24 alleles (Table 2).

The polymorphisms that lead to an amino acid substitution were G37V, T110P, G127S, M137I, I142M/I142T, N146S, R154H, P168Q, T194P, R211Q, Q215R, Q222K, and S240P. In addition, two silent mutations were observed at codons P42P (CCG/CCA) and S138S (AGC/AGT), but new variants compared to those already known in the literature were not observed in the analyzed sequences.

The allele frequencies reported in Table 3 allow the observation that the allele 2 is the most frequent in all the populations/breeds, except for the AL (12.96%) breed, reaching the highest frequency in TE (72.92%).

High frequencies were observed also for the allele 1 with frequencies between 8.33% (TE) and 48.15% (AL); the allele 3 is the third most frequent. In particular, this allele is present in cosmopolitan breeds with a low percentage (9.26% in AL and 2.63% in SA), but it is rather frequent in the Italian native goats with a range between 4.17% (BM) and 12.50% (CP, GA). Further alleles with a frequency equal or higher of 5% are moreover observed in BM (4), CP (5, 6, 10, 14, 19), GC (5, 14), GM (13, 22, 23), AL (4, 8, 17), and SA (4, 8, 18). Regarding the alleles common to several populations/breeds, the following have to be considered: allele 5 (BM, CP, FV, FL, GC, TE) and allele 14 (BM, CP, FL, GC, TE). BM is the more variable; indeed, 12 out of 24 alleles have frequencies different from zero. 

**Figure 1:**
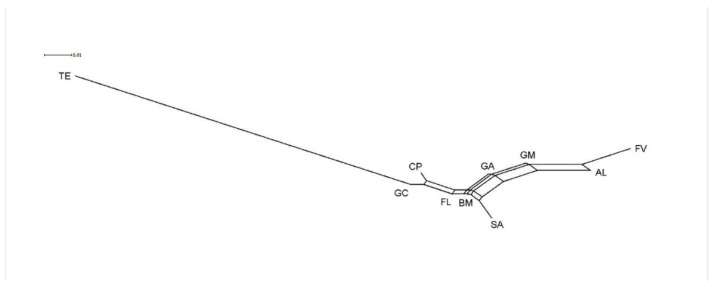
Pairwise *F_ST_* genetic distance tree between the goat population/breeds (eight from Central and Southern Italy and two cosmopolitan goat breeds).BM: Bianca Monticellana; CP: Capestrina; FV: Facciuta della Valnerina; FL: Fulva del Lazio; GA: Garganica; GC: Grigia Ciociara; GM: Grigia Molisana; TE: Teramana; AL: Alpine; SA: Saanen.

The AMOVA analysis shows little variability among populations/breeds (5.83%) and a great variability within populations/breeds (94.17%), as estimated by the highly significance of the global *F*_ST_ (0.058- *p* < 0.001). This situation can be explained by the data already discussed in Table 3, where it is evident that in all the genetic types, more than 60% of chromosomes belonged to one of the three most frequent alleles. The obtained results are confirmed by the genetic distance tree represented in Figure 1, where it has to be outlined that the position of the TE breed is the most distant from the others; this situation could probably be due to the high frequency of the allele 2. Moreover, Figure 1 shows that the populations/breeds from the Lazio region (BM, GC, CP, FL) are close to each other and close to GA; the cosmopolitan breeds (mainly SA) are quite close to these populations/breeds, probably as a consequence of the same genetic introgression. In addition, FV also takes a position quite isolated from the others, except AL.

## 4. Discussion

To date, polymorphisms giving rise to the amino acid substitutions associated with resistance to scrapie in goats were investigated by many authors, and some of those SNPs were found in the populations/breeds studied in this research. In particular, S127 [23], able to extend the incubation time, was detected in alleles 12 present in BM (2.08%) and 13, which is present in BM (2.08%) and GM (8.33%). Regarding the M142 allele polymorphism [24,25], in the same way associated to an increase of the incubation time, it was found in alleles 8 (AL 11.11%, SA 5.26%), 16 (BM 2.08%), 21 (GC 2.08%), and 24 (TE 2.08%) in low percentages.

The H154 allele, associated with a delayed progression of the disease [26,29,35], known as a risk factor for atypical scrapie [36], was detected in allele 6, found in BM (2.08%), CP (7.50%), GA (4.17%), and in GM (2.78%); in allele 20 found in CP (2.50%); and in allele 21 found in GC (2.08%).

On the other hand, K222 allele, which confers resistance against classical scrapie [25,29,30,33,41], was detected in two alleles; the allele 3 was present in all populations/breeds, while the allele 18 was observed in CP (2.50%) and SA (5.26%). In addition, S146 allele, which explicates a strong protective effect against classical scrapie, was found in Cypriot indigenous goats [27,28,54] and in Damascus goats bred in Greece (c.a 6.0% in all cases) [49]. The S146 allele was present in Anatolian Black and Kilis goats with frequencies of 3% and 5%, respectively [55], and the frequency of the protective variant in three of the main Ethiopian goat breeds was observed in both homozygous and heterozygous combinations [56]. In our study, S146 was found in allele 7 present only in AL with a frequency of 3.70%, so it could be hypothesized that this breed is quite prone to being resistant. 

The Q211 allele, which seems to prolong the incubation time [57], is present in allele 4, which was detected in BM (6.25%), AL (5.56%), and SA (23.68%).

Maestrale et al. [41] recorded a longer incubation time for scrapie also in the presence of allelic variants Q168 and P240. The first one is present in alleles 5 (BM: 4.17%; CP: 5.00%; FV: 2.78%; FL 4.76%, GC: 6.25%; TE: 2.08%) and 19 (CP: 5.00%); the second one is present in 12 alleles with frequencies similar to S240 (present in the other 12 alleles), but its role in scrapie susceptibility still remains controversial [40]. No direct effects on scrapie have been reported for the other observed alleles (G37V, T110P, M137I) [21,29,57].

Furthermore, double polymorphisms on codon 137 and 142 for allele 16, on codon 142 and 154 for allele 21, and on codon 110 and 142 for allele 24 were detected in a heterozygous form in the animals of our study, even if in low percentages, and these alleles were included in PHASE 2.1 output (using an inferential statistical approach) but with a very little probability of existence. Obviously, sequencing alone does not allow determination of whether double mutations are in phase or in repulsion at the same chromosomal locus and further tests could be carried out. While a subsequent investigation of the very interesting Alpine breed samples carrying the K222 and S146 variants as markers of resistance was performed, for the other populations/breeds, the coexistence of more than one mutant allele in heterozygous form was not verified by cloning and further sequencing, so we cannot state if these alleles are in phase or in repulsion at the chromosomal locus. The reason is because this investigation was not the first instance aim of the research, but it is not excluded that it will be a future object of in-depth analysis.

According to EFSA recommendations [39], the ranking of resistant alleles is: K222 > D146 = S146 > Q211 = H154 = M142 > S127 = H143 > wild type. Animals characterized by K222, D146, and S146 alleles or genotypes are to be considered very interesting as carriers of resistance markers, also in accordance with EU Regulation 772/2020. Particularly, animals carrying the K222 allele at various frequencies (min 0.04 in BM, max 0.15 in CP) are present in all the analyzed goat populations/breeds. For this purpose, the presence in the lpine breed of two subjects heterozygous at position 146 (N/S) and 222 (Q/K) led to further analysis by cloning and sequencing, which revealed that the S146 and K222 variants were in repulsion. In addition, concerning the genetic distance tree, the position of FV, quite far away from the others except for AL, could be explained by a possible introgression between FV and AL. Furthermore, these results, in particular about the position of TE and the proximity among other genetic types, are supported by similar data obtained through a different statistical approach in a previous study [58]. All results obtained about the allele variants frequencies may be useful for the design and implementation of a genetic selection plan based on SNPs genotyping [59], similar to what has been done in the breeding programs already established and applied to sheep [14]. Genetic selection plans could be useful to allow a decrease in the number of animals culled, for example, during scrapie outbreaks, significantly reducing the related costs. However, mostly, it is relevant to preventing scrapie diffusion and outbreak onset. As highlighted also by Regulation 772/2020, considering also the low frequency of the alleles of interest, it is very important to prevent the selective pressure that could exert a negative effect on genetic diversity and on variable genotype/phenotype traits. Furthermore, goat farmers should be encouraged to use these animals as breeders in their farms under the supervision of national breeders associations. Before the application of any measure, it is necessary to determine possible adverse effects of selection towards scrapie resistance on other important animal traits and fully understand the allelic interactions in the involved codons. At the same time, caution is mandatory since unidimensional selection towards enhancing scrapie resistance could potentially lead to a reduction of genetic variability or biodiversity and to increased inbreeding, especially given the relatively low population frequencies of resistance-associated alleles [49]. In other words, a breeding program based on scrapie resistance has to be considered with the dual aim of increasing the low frequency of the classical scrapie resistance alleles in Italian goat populations/breeds and, at the same time, of preventing high levels of inbreeding in order to maintain good genetic variability especially in endangered populations/breeds.

Moreover, it is possible to observe that there is no noticeable difference between the strongly selected breed SA and the other native not selected populations/breeds in terms of resistance to scrapie; at the present time, the only breed that shows S146 and K222 alleles is in fact the cosmopolitan AL breed.

## 5. Conclusions

This work highlights that the investigated SNPs are not ubiquitously present in all the analyzed Italian goat populations/breeds and that their frequencies are different from each other; anyway, the K222 allele is present in all the populations/breeds.

Taking into account also the Regulation EU 772/2020 in force, these data are a good basis to adopt scrapie eradication strategies based on genetic selection. The success achieved by the breeding programs for scrapie eradication applied to sheep represents a robust strategy to follow and adopt also in goats, where the K222 polymorphism also has the advantage of reducing susceptibility to BSE. There is indeed a large amount of experimental and in-field data relative to this allele, demonstrating and supporting a strong association with resistance to classical scrapie. On the other hand, to date, for the S146 allele, collected data indicate a strong resistance to classical scrapie but not as strong as that of allele K222. Due to the very low frequencies of alleles D146 and S146 in the rest of Europe, no data from field studies regarding resistance to other European scrapie strains are available [39]. On this basis, an allele containing the S146 variant detected in this study represents interesting data.

In order to better understand the role of all the known and unknown polymorphisms in resistance/susceptibility to the classical and atypical forms of the disease, scrapie infection studies and case reports are still necessary, also in these populations/breeds. Indeed, it is also important to establish whether these mutations have effects, either alone or in association with others.

## Figures and Tables

**Table 1:** Allele frequencies of *PRNP* polymorphisms (given as a fraction of unit) in the studied populations/breeds (highlighted in grey: variants). The number of animals is reported in brackets.

Codon Position	Codon Sequence	aa	Populations/Breeds										
			BM (24)	CP (20)	FV (18)	FL (21)	GA (24)	GC (24)	GM (18)	TE (24)	AL (27)	SA (19)	TOT (219)
37	GGG	G	0.98	1.00	1.00	0.95	0.96	1.00	0.92	1.00	1.00	1.00	0.98
	GTG	V	0.02	-	-	0.05	0.04	-	0.08	-	-	-	0.02
110	ACC	T	0.96	0.90 *	1.00	0.95	1.00	0.88	0.86	0.94	1.00	1.00	0.95
	CCC	P	0.04	0.10	-	0.05	-	0.12	0.14	0.06	-	-	0.05
127	GGC	G	0.96	1.00	1.00	1.00	1.00	1.00	0.92	1.00	1.00	1.00	0.99
	ACG	S	0.04	-	-	-	-	-	0.08		-	-	0.01
137	ATG	M	0.96	0.90	1.00	1.00	1.00	0.98	1.00	1.00	1.00	1.00	0.98
	ATA	I	0.04	0.10	-	-	-	0.02	-	-	-	-	0.02
142	ATA	I	0.98	1.00	1.00	1.00	1.00	0.98	1.00	0.98	0.77 *	0.95	0.96 *
	ATG	M	0.02	-	-	-	-	0.02	-	0.02	0.13	0.05	0.03
	ACC	T	-	-	-	-	-	-	-	-	0.10	-	0.01
146	AAT	N	1.00	1.00	1.00	1.00	1.00	1.00	1.00	1.00	0.96	1.00	1.00
	AGT	S	-	-	-	-	-	-	-	-	0.04	-	-
154	CGT	R	0.98	0.90	1.00	1.00	0.96	0.98	0.97	1.00	1.00	1.00	0.98
	CAT	H	0.02	0.10	-	-	0.04	0.02	0.03	-	-	-	0.02
168	CCA	P	0.96	0.90	0.97	0.95	1.00	0.94	1.00	0.98	1.00	1.00	0.97
	CAA	Q	0.04	0.10	0.03	0.05	-	0.06	-	0.02	-	-	0.03
194	ACC	T	1.00	1.00	1.00	1.00	1.00	1.00	1.00	1.00	1.00	1.00	1.00
	CCC	P	-	-	-	-	-	-	-	-	-	-	-
211	CGA	R	0.94	1.00	1.00	1.00	1.00	1.00	1.00	1.00	0.94	0.76	0.97
	CAA	Q	0.06	-	-	-	-	-	-	-	0.06	0.24	0.03
215	CAA	Q	1.00	1.00	1.00	1.00	1.00	1.00	1.00	1.00	1.00	1.00	1.00
	CGA	R	-	-	-	-	-	-	-	-	-	-	-
222	CAG	Q	0.96	0.85	0.92 *	0.88	0.87	0.90	0.92	0.90	0.91	0.92	0.90
	AAG	K	0.04	0.15	0.08	0.12	0.13	0.10	0.08	0.10	0.09	0.08	0.10
240	CCC	S	0.50 *	0.63 *	0.39 *	0.50 *	0.58 *	0.50	0.44	0.25	0.52	0.53 *	0.49 *
	TCC	P	0.50	0.37	0.61	0.50	0.42	0.50	0.56	0.75	0.48	0.47	0.51

BM: Bianca Monticellana; CP: Capestrina; FV: Facciuta della Valnerina; FL: Fulva del Lazio; GA: Garganica; GC: Grigia Ciociara; GM: Grigia Molisana; TE: Teramana; AL: Alpine; SA: Saanen; aa: amino acid. * not in HW equilibrium; *p* ≤ 0.05. -: not detected in population/breeds (frequency of 0); underlined aa: alleles known as resistance markers.

**Table 2:** *PRNP* alleles estimated by PHASE 2.1 in caprine populations/breeds.

*PRNP* Alleles	Codon Position												
	37	110	127	137	142	146	154	168	194	211	215	222	240
1	G	T	G	M	I	N	R	P	T	R	Q	Q	S
2	G	T	G	M	I	N	R	P	T	R	Q	Q	P
3	G	T	G	M	I	N	R	P	T	R	Q	K	S
4	G	T	G	M	I	N	R	P	T	Q	Q	Q	S
5	G	T	G	M	I	N	R	Q	T	R	Q	Q	P
6	G	T	G	M	I	N	H	P	T	R	Q	Q	S
7	G	T	G	M	I	S	R	P	T	R	Q	Q	P
8	G	T	G	M	M	N	R	P	T	R	Q	Q	P
9	G	T	G	M	T	N	R	P	T	R	Q	Q	S
10	G	T	G	I	I	N	R	P	T	R	Q	Q	S
11	G	T	G	I	I	N	R	P	T	R	Q	Q	P
12	G	T	S	M	I	N	R	P	T	R	Q	Q	S
13	G	T	S	M	I	N	R	P	T	R	Q	Q	P
14	G	P	G	M	I	N	R	P	T	R	Q	Q	S
15	V	T	G	M	I	N	R	P	T	R	Q	Q	S
16 *	G	T	G	I	M	N	R	P	T	R	Q	Q	P
17	G	T	G	M	T	N	R	P	T	R	Q	Q	P
18	G	T	G	M	I	N	R	P	T	R	Q	K	P
19	G	T	G	M	I	N	R	Q	T	R	Q	Q	S
20	G	T	G	M	I	N	H	P	T	R	Q	Q	P
21 *	G	T	G	M	M	N	H	P	T	R	Q	Q	S
22	G	P	G	M	I	N	R	P	T	R	Q	Q	P
23	V	T	G	M	I	N	R	P	T	R	Q	Q	P
24 *	G	P	G	M	M	N	R	P	T	R	Q	Q	S

G: glycine, V: valine, T: threonine, M: methionine, I: isoleucine, H: histidine, N: asparagine, R: arginine, P: proline, Q: glutamine, S: serine, K: lysine. *: the presence at the same chromosomal locus of double polymorphisms for the alleles 16, 21 and 24 is a probabilistic event as output of PHASE 2.1, but it was not experimentally verified in populations/breeds object of the study.

**Table 3:** *PRNP* allele frequencies (%) estimated by PHASE 2.1 in the individual goat populations/breeds. The number of animals is indicated in brackets.

*PRNP* Alleles	BM (24)	CP (20)	FV (18)	FL (21)	GA (24)	GC (24)	GM (18)	TE (24)	AL (27)	SA (19)
1	29.17	17.50	30.56	28.57	37.50	25.00	33.33	8.33	48.15	26.32
2	39.58	27.50	58.33	45.24	41.67	41.67	25.00	72.92	12.96	36.84
3	4.17	12.50	8.33	11.90	12.50	10.42	8.33	10.42	9.26	2.63
4	6.25	-	-	-	-	-	-	-	5.56	23.68
5	4.17	5.00	2.78	4.76	-	6.25	-	2.08	-	-
6	2.08	7.50	-	-	4.17	-	2.78	-	-	-
7	-	-	-	-	-	-	-	-	3.70	-
8	-		-	-	-	-	-	-	11.11	5.26
9		-	-	-	-	-	-	-	-	-
10	-	10.00	-	-	-	-	-	-	-	-
11	2.08	-	-	-	-	2.08	-	-	-	-
12	2.08	-	-	-	-	-	-	-	-	-
13	2.08	-	-	-	-	-	8.33	-	-	-
14	4.17	10.00	-	4.76	-	12.50	-	4.17	-	-
15	2.08	-	-	4.76	4.17	-	-	-	-	-
16	2.08	-	-	-	-	-	-	-	-	-
17	-	-	-	-	-	-	-	-	9.26	-
18	-	2.50	-	-		-	-	-	-	5.26
19	-	5.00	-	-	-	-	-	-	-	-
20	-	2.50	-	-	-	-	-	-	-	-
21	-	-	-	-	-	2.08			-	-
22	-	-	-	-	-	-	13.89	-	-	-
23	-	-	-	-	-	-	8.33	-	-	-
24	-	-	-	-	-		-	2.08	-	-
TOT	100	100	100	100	100	100	100	100	100	100

BM: Bianca Monticellana; CP: Capestrina; FV: Facciuta della Valnerina; FL: Fulva del Lazio; GA: Garganica; GC: Grigia Ciociara; GM: Grigia Molisana; TE: Teramana; AL: Alpine; SA: Saanen.

## Data Availability

The data presented in this study are available in this article and related Supplementary Materials.

## Supplementary Materials

The following are available online at https://www.mdpi.com/2076-2615/11/2/333/s1, File S1: Autochthonous Italian Populations/Breeds, Table S2: Sampling of populations/breeds object of this study.
