# Supplementary material for: PRNP Polymorphisms in Eight Local Goat Populations/Breeds from Central and Southern Italy
Source: Animals (Basel). 2021 Jan 28;11(2):333. doi: 10.3390/ani11020333 (PMC7911694; doi:10.3390/ani11020333)
Supplement: Supplementary file 1 [file animals-11-00333-s001.zip › Supplementary File 1.docx]

**Supplementary File 1**

**AUTOCHTHONOUS ITALIAN POPULATIONS/BREEDS**

**Bianca Monticellana (BM)**


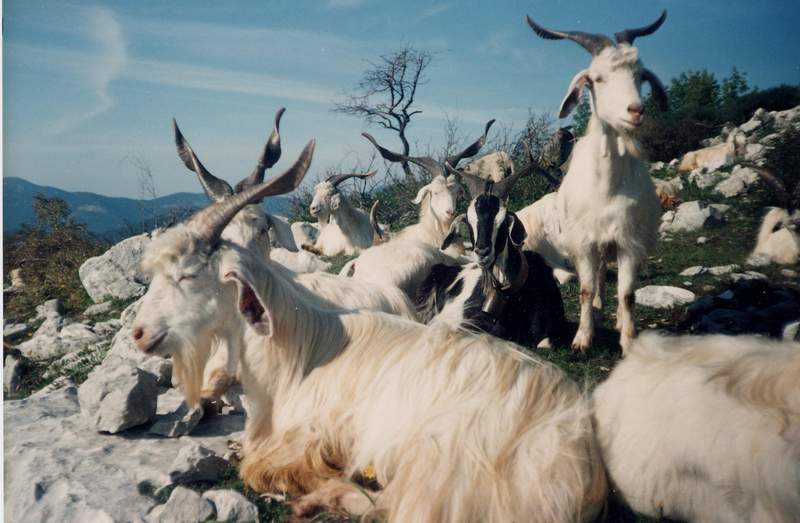
These animals seem to derive from goat type described in Lazio in the early twentieth century as "Bianca Romana" and then not reported and nor census.

**Spread** **The breeding of Monticellana is widespread in areas of Southern Lazio. Many farms are focused on Aurunci and Ausoni Mountains in both sides of Frosinone and Latina provinces.**

**Consistences** In 2014 in the national Herdbook (HB) 1508 animals were enrolled.

**Attitudes and productions The main production is milk, even if kid’s meat product is very appreciated.**

**Typical breed characters Medium-large size in females and large in males are observed. Coat color is white and skin color** **is pinkish-white.** Horns are present in both sexes.

**Capestrina (CP)**


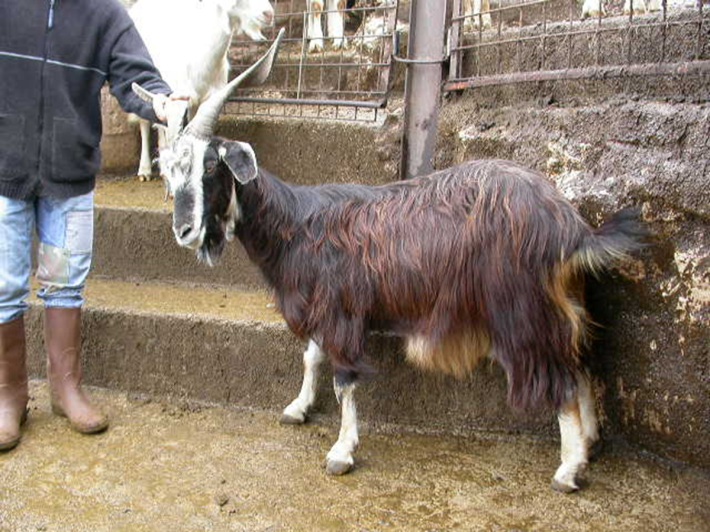
The probable origin area of this goat population is the ridge of the Lepine, Ausoni and Aurunci Mountains (Lazio region).

**Spread** **The breeding area is located in the South of Lazio with the main consistence along the ridges of the provinces of Rome, Frosinone and Latina of the Lepine Mountains, Ausoni and Aurunci. A widespread presence is also found in the Mainarde Group Mountains and in Val Comino.** **Last layers of diffusion seem to be the Prenestini Mountains in the Rome province.**

**Consistences** In 2014 in the national Herdbook (HB) 830 animals were enrolled.

**Attitudes and productions** **The genetic type is distinctly less dairy than the other goat populations of the Lazio region and is generally bred for the production of the kid and milked for a short period (after the sale of this one).**

**Typical breed characters** **Size is large, the coat is usually black or dyed black, tan is uniform throughout the body and limbs are light with frontal black line. There may be changes of the pigment that create brown dorsal filaments or white areas ventral and perianal.** Horns are present in both sexes.

**Fulva del Lazio (Rossa) (FL)**


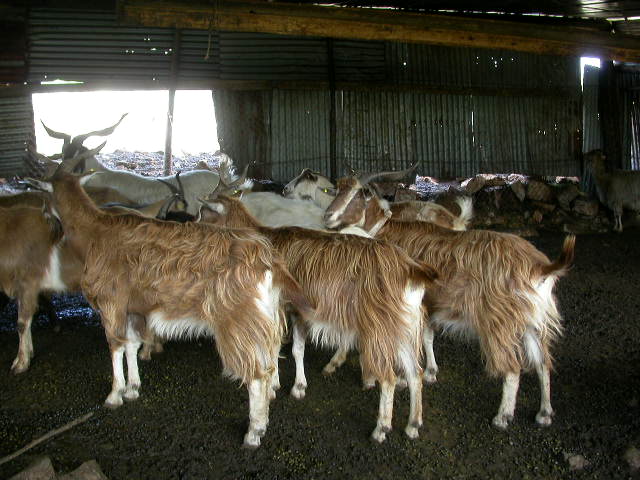
The Fulva seems to be one of the oldest native goats of the Lazio region. Generally is reared with the Bianca Monticellana, Ciociara and Capestrina goats. The ridge of the Lazio mountain is certainly the origin area of this population.

**Spread The distribution area of the breed is located in the South of Lazio with the main consistence in the ridge area of the provinces of Rome and Latina of the Lepine Mountains. Sporadic presence on the Aurunci and Mainarde Mountains.**

**Consistences** The farms where the Fulva goat was detected are about 5 and are among those involved in the census on native goats of Lazio, realized by ARSIAL. The goat is enrolled in a Volunteer Registry maintained by ARSIAL.

**Attitudes and productions The main production is milk even if the "product" kid is very appreciated.**

**Typical breed characters Large size.** **Usually with red coat and tan uniform throughout the body and limbs white with possible frontal black line. This line can be slightly hinted on the dorsal part of the trunk. There may be changes of the pigment that create or brown dorsal filaments or white areas ventral and perianal.** Horns in both sexes.

**Grigia ciociara (GC)**

**
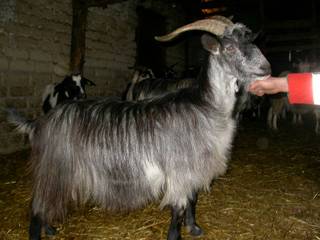
**The Ciociara Grigia or Grigia Ciociara is an indigenous breed of domestic goat from Lazio in central Italy. It takes its name from the Ciociaria, the area around Frosinone. It is thought to have originated in the area of the Monti Aurunci and the Monti Ausoni.

**Spread** It is raised in those mountains, in the Monti Lepini, and in the Val Comino. Because of the transhumant management of the herds it has also diffused into some neighbouring areas of Campania and Abruzzo.

**Consistences** It is one of the forty-three autochthonous Italian goat breeds of limited distribution for which a Herd book is kept by the Associazione Nazionale della Pastorizia, the Italian national association of sheep and goat breeders. At the end of 2013 the registered population was 674.

**Attitudes and productions The main production is milk with typical products ("Marzolina", "Formaggio di Capra" and "Ricotta Secca") even if kid’s meat product is very appreciated.**

**Typical breed characters Size is medium. Coat color is dark grey and light silvery is uniform throughout the body including the limbs and the head where maculation and shade of grey are observed.**

**Facciuta della Valnerina (FV)**


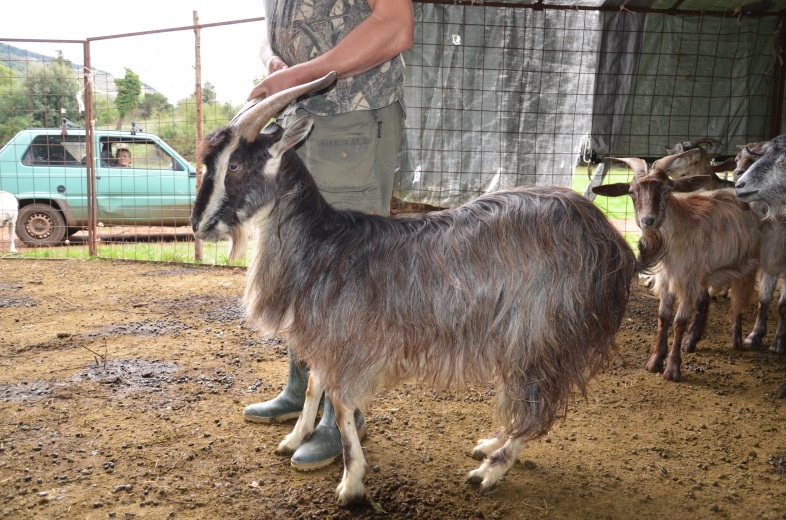
The Valnerina goat is also known with names that refer to its most particular feature or two white lists on the face, and, for this reason, different names are attributed such as “Rigatina", "Facciuta bianca" and "Mascherina."

**Spread The Facciuta goat is typical of the Valnerina area, in the South of the Umbria, but it is also widespread in the Marche region, especially in Vissano and Camerinese, in Sabina and in some areas of Abruzzo.**

**Consistences The current consistency is around 200-300 heads, distributed in the above mentioned areas and it is often bred with other types of "Apennine" goats.** The goat is enrolled in a Volunteer Registry maintained by Umbria region.

**Attitudes and productions** **The breed has a double attitude, but there are some farms which produce only meat.**

**Typical breed characters Size is medium-large. The coat is black and long with reddish hues, while the ends of the limbs are clear (white or beige) as the** **belly and perianal area****; some heads, however, can show a clear coat at the side. Some heads with completely red coat and limbs, belly and perianal area white were also found.** Horns are present in both sexes.

(Work funded under the Fondazione Cassa di Risparmio di Perugia project n. 2013.0208.021 by Department of Agricultural, Food and Environmental Sciences-University of Perugia).

**Capra di Campobasso (Grigia molisana, di Montefalcone) (GM)**


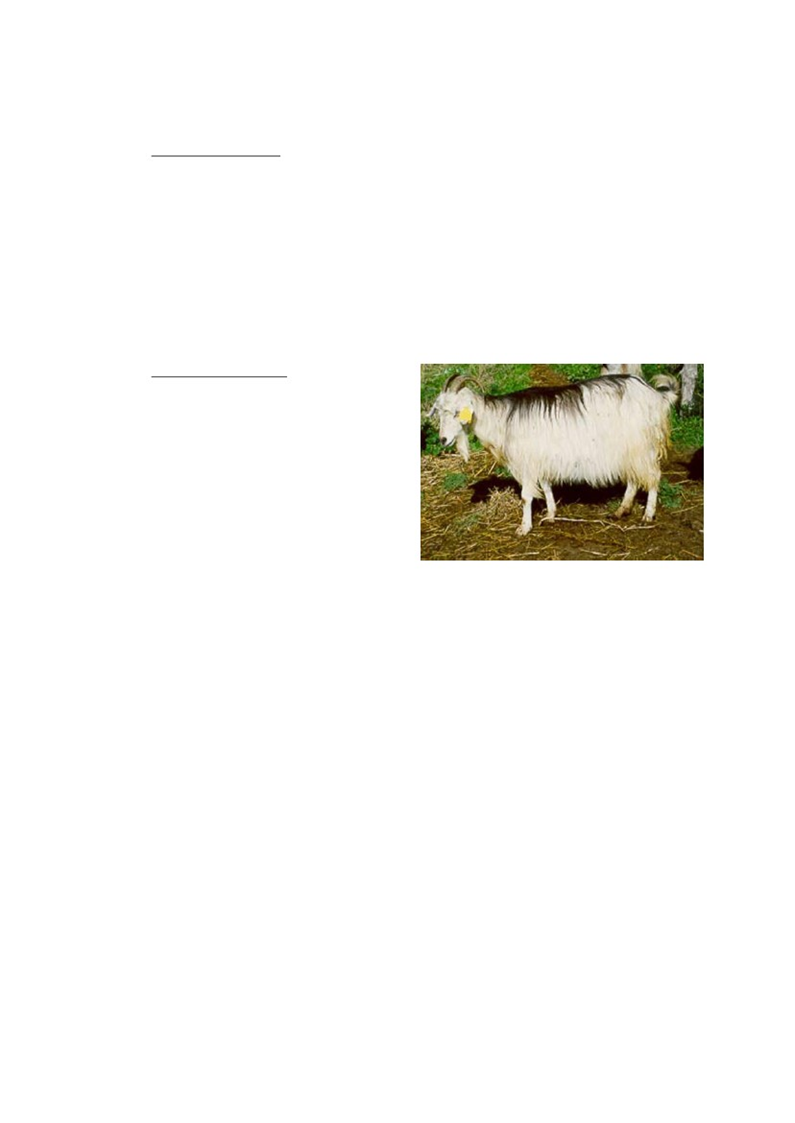
The Montefalcone goat is present throughout the Molise region, particularly between the municipalities of Acquaviva Colle Croci, S. Felice del Molise, Castelmauro and Montemitro, but there is an higher concentration in the municipality of Montefalcone where it is bred according to ancient traditions. Its origin is still to be defined, but it seems to have been influenced by Maltese blood. The breeding system is a semi-wild grazing in woodlands and fallow land.

**Consistences** In 2014 in the national Herdbook (HB) 69 animals in 2 farms were enrolled.

**Attitudes and productions The main productions are milk but also kid’s meat product.**

**Typical breed characters Size is medium.** **The coat is very elegant and usually characterized by different colors including white and black in different proportions, so that in the most common combinations are present animals with white coat characterized by a black dorsal line. The hair is long and flowing.** Horns are present in both sexes.

**Teramana (TE)**


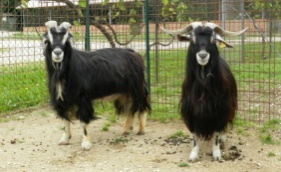
**Spread** The Teramana is an indigenous breed of domestic goat coming from the province of Teramo, in Abruzzo in Southern Italy, and is raised only in that area.

**Consistences** At the end of 2013 in the national Herdbook (HB) 58 animals were enrolled; the breed was listed as endangered by the FAO in 2007.

**Attitudes and productions The breed has a double attitude, but there are some farms which produce only meat.**

**Typical breed characters** Its characteristics are very heterogeneous due to the frequent cross-breeding with the Garganica breed. The coat, usually thick and flowing, is mainly grey with white lists at the head, while the limbs, the belly and the under tail are white. There are black longitudinal striations in the cranial and dorsal parts of the four limbs. The head is elongated with straight profile and is endowed in both sexes with divergent lyre or sickle horns; the beard and tuft are present at the most developed synchypite in the male. The ears are medium long or short, usually horizontal and never completely hanging.

**Garganica (GA)**


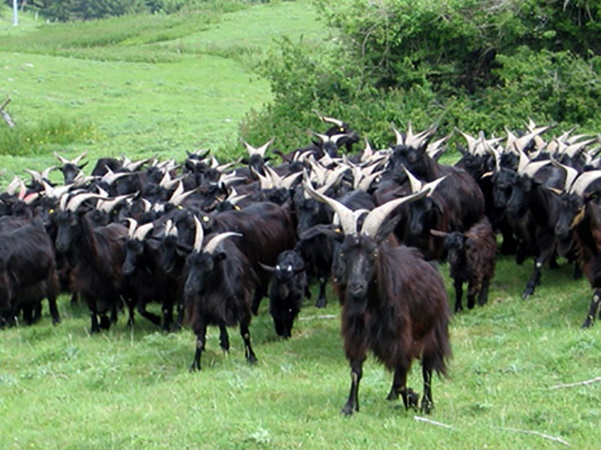
**Spread** The Garganica is indigenous to the Gargano, and derives from cross-breeding of local animals with goats imported from western Europe, probably at the same time as consequence of the importation of Merino sheep that led to the formation of the Gentile di Puglia sheep breed.

The Garganica is one of the eight autochthonous Italian goat breeds for which a genealogical herdbook is kept by the *“Associazione Nazionale della Pastorizia”*, the Italian national association of sheep-breeders.

**Consistences** The breed standard was approved in 1976. The conservation status of the breed was listed as "endangered" by the FAO in 2007. In 2008 the total population was estimated at 3000, of which 511 were registered in the Herd book. At the end of 2013 the registered population was 773.

**Attitudes and productions The milk yield of the Garganica is reported as 120 litres in 180 days for primiparous nannies, and 170 l in 210 days for pluriparous ones, in one source; another gives a yield of 180–250 l. The milk is used to make the traditional regional cheeses of the Gargano, including *“canestrato”* and *“cacioricotta del Gargano”.* The meat is used to make *“muscisca”*, a traditional food of transhumant pastoralists. Strips of goat's meat are salted, flavoured with garlic, and air- or sun-dried.**

**Typical breed characters The animals are of medium height and not heavy: adult males typically weigh 65 kg and females 50 kg. The heads are rather small and adults have long, coarse black or dark chestnut hair. The skin of the kids are prized for their curly, raven-black hair. The breed is noted for its toughness and is well suited to being raised in a wild state in very difficult habitats.**

**GEOGRAPHICAL DISTRIBUTION**


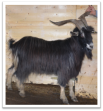


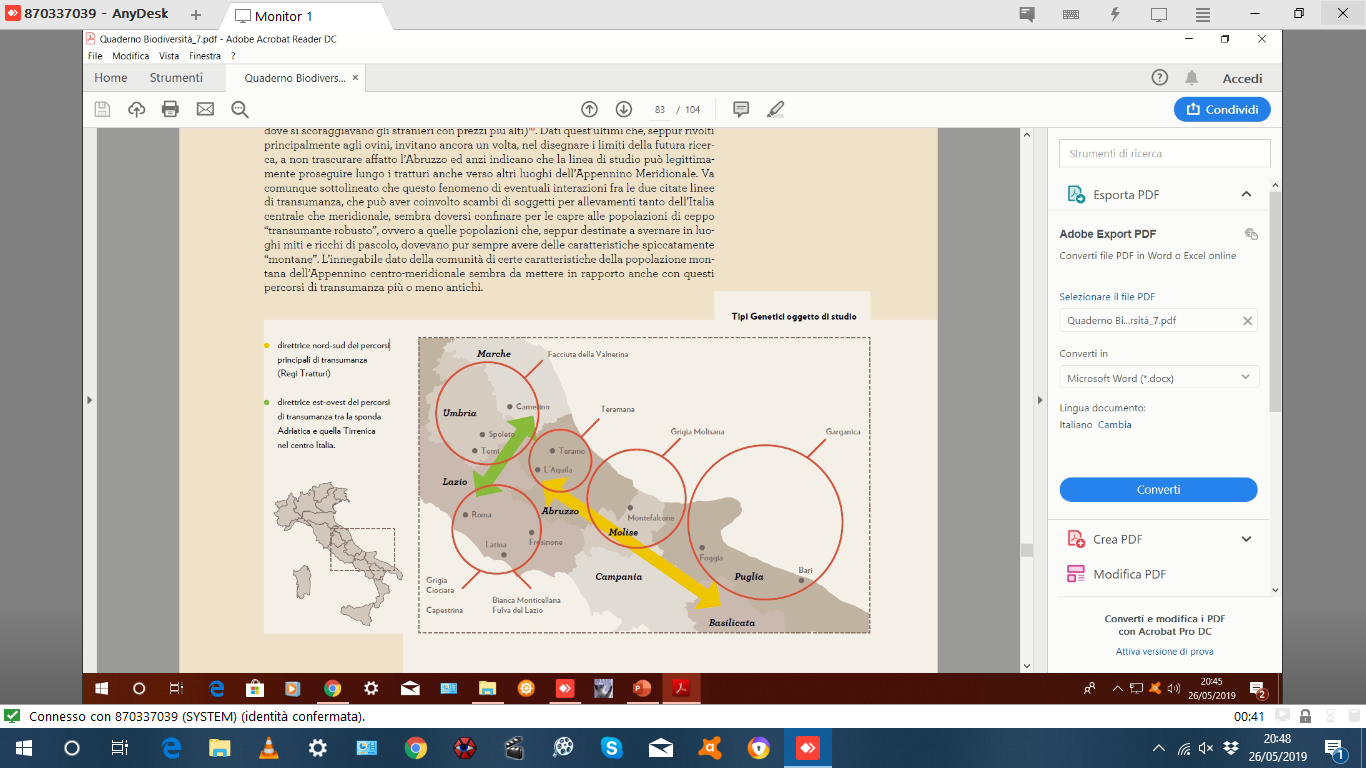

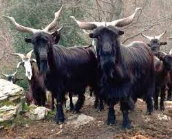

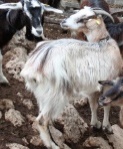

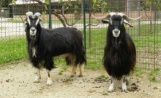


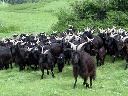

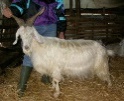


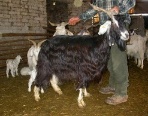


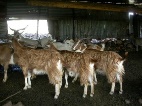


**COSMOPOLITAN BREEDS**

**Alpine (Camosciata delle Alpi) (AL)**

Native to Switzerland, Alpine has spread in many European countries, mainly in France, and also outside Europe. In Italy it has its greatest consistency in the regions of the Alps, with significant presences in the rest of the national territory.
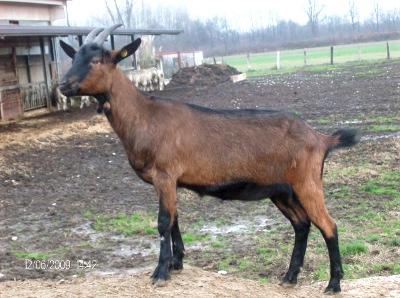
 The Italian Herd Book was activated in 1973.

Coat is fawn, in various shades, with reel line and ends of the limbs and black claws, characteristic facial mask, hair uniformly short and fine; thin skin pigmented in black. Coats with uneven colouration are tolerated (in particular white spots, slits on the muzzle) or dark cloaks tending to black. Size is medium big. Head is relatively small, light and fine; fronto-nasal profile is straight; beard is present especially in the males; long ears are carried sideways, obliquely and forward (never abandoned and hanging); tuft of ruffled hair in the frontal zone are present only in the males; it is possible to observe horns in both males and females.

**Attitudes and productions** The main production is milk.

**Saanen (SA)**


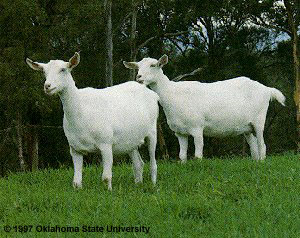
The Saanen dairy goat originated in Switzerland, in the Saanen Valley. Saanen are heavy milk producers and usually yield 3-4 percent milk fat. It is medium to large in size (weighing approximately 145 lbs/65kg) with rugged bone and plenty of vigor. Saanens are white or light cream in colour, mainly withe. Spots on the skin are not discriminated against. Small spots of colour on the hair are allowable, but not desirable. The hair should be short and fine, although a fringe over the spine and thighs is often present. Ears should be erect and alertly carried, preferably pointing forward. The face should be straight or dished. A tendency toward a roman nose is discriminated against. The breed is sensitive to excessive sunlight and it performs best in cooler conditions. The provision of shade is essential and tan skin is preferable.

**Attitudes and productions** The main production is milk.

***References***

Asso.na.pa. Associazione Nazionale della Pastorizia (Italian National Association of Sheep and Goat Breeders) http://www.assonapa.it/norme_ecc/CAPRINI-RA_Standard_WEB/

Daniele Bigi, Alessio Zanon (2008). *Atlante delle razze autoctone: Bovini, equini, ovicaprini, suini allevati in Italia* (in Italian). Milan: Edagricole. [ISBN](https://en.wikipedia.org/wiki/ISBN_(identifier)) [9788850652594](https://en.wikipedia.org/wiki/Special:BookSources/9788850652594).

https://www.capre.it/le-razze-in-italia/libro-genealogico-caprino/10-camosciata-delle-alpi-o-alpine.html

http://afs.okstate.edu/breeds/goats/saanen/index.html/
