# Supplementary material for: PRNP Polymorphisms in Eight Local Goat Populations/Breeds from Central and Southern Italy
Source: Animals (Basel). 2021 Jan 28;11(2):333. doi: 10.3390/ani11020333 (PMC7911694; doi:10.3390/ani11020333)
Supplement: Supplementary file 1 [file animals-11-00333-s001.zip › Supplementary Table 1.docx]

Supplementary Table 1. Sampling of populations/breeds object of this study

| **POPULATIONS/BREEDS** | **FARM** | **SAMPLES NUMBER** |
| --- | --- | --- |
| GRIGIA CIOCIARA (GC) | 1 | 5 |
|  | 2 | 6 |
|  | 3 | 5 |
|  | 4 | 3 |
|  | 5 | 5 |
| BIANCA MONTICELLANA (BM) | 1 | 6 |
|  | 2 | 2 |
|  | 3 | 4 |
|  | 4 | 6 |
|  | 5 | 6 |
| CAPESTRINA (CP) | 1 | 1 |
|  | 2 | 1 |
|  | 3 | 3 |
|  | 4 | 4 |
|  | 5 | 1 |
|  | 6 | 10 |
| FULVA DEL LAZIO (FL) | 1 | 1 |
|  | 2 | 6 |
|  | 3 | 14 |
| FACCIUTA DELLA VALNERINA (FV) | 1 | 1 |
|  | 2 | 2 |
|  | 3 | 1 |
|  | 4 | 2 |
|  | 5 | 2 |
|  | 6 | 2 |
|  | 7 | 2 |
|  | 8 | 3 |
|  | 9 | 3 |
| TERAMANA (TE) | 1 | 16 |
|  | 2 | 8 |
| GARGANICA (GA) | 1 | 5 |
|  | 2 | 3 |
|  | 3 | 3 |
|  | 4 | 2 |
|  | 5 | 2 |
|  | 6 | 4 |
|  | 7 | 2 |
|  | 8 | 3 |
| GRIGIA MOLISANA (GM) | 1 | 10 |
|  | 2 | 8 |
| **COSMOPOLITAN BREEDS** | **FARM** | **SAMPLES NUMBER** |
| ALPINE (AL) | 1 | 10 |
|  | 2 | 17 |
| SAANEN (SA) | 1 | 8 |
|  | 2 | 11 |
